# Comparison of Health Information Technology Use Between American Adults With and Without Chronic Health Conditions: Findings From The National Health Interview Survey 2012

**DOI:** 10.2196/jmir.6989

**Published:** 2017-10-05

**Authors:** Yan Zhang, Romy Lauche, David Sibbritt, Bolanle Olaniran, Ronald Cook, Jon Adams

**Affiliations:** ^1^ Division of Integrative Medicine Department of Family and Community Medicine, School of Medicine Texas Tech University Health Sciences Center Lubbock, TX United States; ^2^ Australian Research Centre in Complementary and Integrative Medicine Faculty of Health University of Technology Sydney Sydney Australia; ^3^ Department of Communication Studies College of Media and Communication Texas Tech University Lubbock, TX United States; ^4^ Department of Family and Community Medicine School of Medicine Texas Tech University Health Sciences Center Lubbock, TX United States

**Keywords:** health information technology, chronic illness

## Abstract

**Background:**

Health information technology (HIT) is utilized by people with different chronic conditions such as diabetes and hypertension. However, there has been no comparison of HIT use between persons without a chronic condition, with one chronic condition, and multiple (≥2) chronic conditions (MCCs).

**Objective:**

The aim of the study was to assess the difference in HIT use between persons without a chronic condition, with one chronic condition, and with MCCs, to describe the characteristics of HIT use among those with chronic conditions and to identify the predictors of HIT use of the persons with one chronic condition and MCCs.

**Methods:**

A secondary data analysis was conducted in spring 2017 using the National Health Interview Survey (NHIS) 2012 Family Core and Sample Adult Core datasets that yielded 34,525 respondents aged 18 years and older. Measures included overall HIT use (ie, any use of the following five HIT on the Internet: seeking health information, ordering prescription, making appointment, emailing health provider, and using health chat groups), as well as sociodemographic and health-related characteristics. Sociodemographic and health characteristics were compared between HIT users and nonusers among those who reported having at least one chronic condition using chi-square tests. Independent predictors of HIT use were identified using multiple logistic regression analyses for those with one chronic condition, with MCCs, and without a chronic condition. Analyses were weighted and performed at significance level of .005.

**Results:**

In 2012, adults with one health chronic condition (raw count 4147/8551, weighted percentage 48.54%) was significantly higher than among those with MCCs (3816/9637, 39.55%) and those with none of chronic condition (7254/16,337, 44.40%, *P*<.001). Seeking health information was the most prevalent HIT use. Chi-square tests revealed that among adults with chronic conditions, those who used HIT were significantly different from their counterpart peers who did not use HIT in terms of sociodemographic and health characteristics (*P*<.001). Overall, the significant factors related to HIT use were similar among the adults with one chronic condition, with MCCs, or without a chronic condition: younger age, female sex, non-Hispanic white, higher education level, and higher income level were shown to be positively related to the HIT use.

**Conclusions:**

This study provides a snapshot of HIT use among those with chronic conditions and potential factors related to such use. Clinical care and public health communication efforts attempting to leverage more HIT use should acknowledge differential HIT usage as identified in this study to better address communication inequalities and persistent disparities in socioeconomic status.

## Introduction

According to the 2012 update of National Health Interview Survey (NHIS) data [1], among the noninstitutionalized, civilian US adult population, approximately half (117 million) of US adults have at least one of 10 chronic conditions (eg, hypertension, coronary heart disease, stroke, diabetes, and cancer). More specifically, 24.3% report 1 chronic condition, 13.8% report 2 chronic conditions, and 11.7% report 3 or more chronic conditions, which indicates that around 1 in 4 American adults have multiple (≥2) chronic conditions (MCCs).

The use of health information technology (HIT) can include a wide range of activities, from searching general health information to using individual computerized modules or Web portals. HIT has been utilized by people with different specific chronic conditions such as diabetes [2,3] and hypertension [4]. Five HIT uses measured in NHIS include seeking Web-based health information, ordering a Web-based prescription, scheduling a Web-based appointment, communicating with a health care provider over email, or using Web-based chat groups to learn about health topics. On the basis of NHIS 2009 and 2011 data, of all the five HIT uses, seeking health information was 7 to 14 times more likely to occur than the other HIT activities among American adults [5]. Other national surveys reported increasing trend of those other HIT activities when compared with their use in the past two decades [6-8]. Literature revealed that the general HIT users tend to be young, women, white, with a relatively higher education level, and a higher income level [9-11]. However, there has been no comparison of HIT use between persons without a chronic condition, with one chronic condition, and with MCCs.

To address this research gap, we analyzed NHIS 2012 data to (1) assess whether patterns of HIT use differ for persons without a chronic condition, with one chronic condition, and with MCCs; (2) describe the characteristics of HIT use among those with chronic conditions; and (3) identify predictors of HIT use among individuals with one chronic condition and MCCs. The aim of this study was to provide health professionals with a better understanding of HIT use among patients with one or more chronic conditions to facilitate better clinical care and patient education.

## Methods

### Study Design

This paper reports a secondary analysis of data from the NHIS, a cross-sectional household interview survey targeting the noninstitutionalized civilian population of the United States conducted by the Centers for Disease Control and Prevention’s (CDC) National Center for Health Statistics (NCHS) periodically. This study utilized the 2012 NHIS Family Core and Sample Adult Core. The NHIS Family Core questionnaire contained information on the participant’s sociodemographic characteristics and health status. Data on chronic conditions and computer use were collected via the Sample Adult Core questionnaire. Details of the NHIS sampling are reported elsewhere [12]. In brief, the interviewed sample consisted of 42,366 eligible households, which yielded 34,525 respondents aged 18 years and older with a final response rate of 79.7%. We retrieved the dataset and performed the analyses in spring 2017.

### Measures

#### Use of Health Information Technology (HIT)

Participants were asked whether they have ever used computers in the past 12 months for any of the following tasks: (1) to look up health information on the Internet (referred as seeking Web-based health information in the text below), (2) to fill a prescription (referred as ordering a Web-based prescription in the text below), (3) to schedule a Web-based appointment with a health care provider, (4) to communicate with a health care provider by email, or (5) to use online chat groups to learn about health topics (referred as using Web-based chat group in the text below). If an individual indicated use for any of these five purposes, they were considered to have used HIT in the past 12 months.

#### Chronic Conditions

The chronic conditions included in this study were 10 most frequently reported physical health conditions from a list of 20 conditions identified by the US Department of Health and Human Services (DHHS) to foster a more consistent and standardized approach to measuring the occurrence of chronic conditions in the United States [13]. Participants were identified as having 1 of the 10 conditions if they have ever been told by a doctor or health care provider that they had hypertension, coronary heart disease, stroke, diabetes, cancer, arthritis, hepatitis, experienced weak or failing kidneys during the past 12 months, asthma, or chronic obstructive pulmonary disease (COPD). COPD was assessed by using responses from 2 survey questions asking adults whether they had ever had emphysema or chronic bronchitis in the past 12 months; adults answering yes to either question were identified as having COPD. Adults who reported having 2 or more chronic conditions were defined as having MCCs.

#### Sociodemographic Characteristics

HIT use has been found to vary by age [12,14], sex [6,15], race or ethnicity [10,16], education level [6,16], employment, marital relationship, and income level [11]. To account for the variations, we included the following sociodemographic data in the analysis: sex (male or female), age (18-29, 30-39, 40-49, 50-64, 65-74, and 75+ years), race or ethnicity (Hispanic, non-Hispanic white, non-Hispanic black, non-Hispanic Asian, and non-Hispanic other), educational attainment (less than high school, high school graduate or some college, Bachelor’s degree, Master’s degree or higher), employment status (not employed in the past 12 months or employed in the past 12 months), annual household income (less than US $15,000, US $15,000-34,999, US $35,000-54,999, US $55,000-74,999, and US $75,000 or more), and marital status (not in relationship or in relationship).

#### Health-Related Characteristics

Previous research suggests that after controlling for sociodemographic characteristics, self-rated health status may not be significantly associated with HIT use [17]. To examine whether this is also true in the population with chronic conditions, we included factors such as general health status (poor or fair, good, and very good or excellent) and body mass index (BMI; <18.5, 18.5-24.9, 25-29.9, or 30 or above) in our analysis.

### Statistical Analyses

Analyses were performed using the Statistical Package for Social Sciences (SPSS) software (IBM SPSS Statistics for Windows, release 24.0. Armonk, NY: IBM Corp). Because NHIS is a complex survey using a multistage probability complex sampling design that incorporates stratification, clustering, and oversampling of some subpopulations (eg, black, Hispanic, and Asian), sampling weights must be used to produce representative estimates and standard errors. We utilized SPSS Complex Samples to compute statistics and standard errors from complex sample designs by incorporating sample designs into survey analysis. HIT use by respondents with and without chronic conditions as well as characteristics of HIT users and nonusers were compared among those who reported having at least one chronic condition, using chi-square tests. Independent predictors of HIT use were identified using multiple logistic regression analyses for those with one chronic condition, with MCCs, and without a chronic condition. All variables were included in the logistic regression analyses without forward or backward procedures. Due to the large sample size, a statistical significance level of .005 was chosen, and the 99.5% CI were calculated.

## Results

### Prevalence of Health Information Technology Use and Chronic Condition Status

In 2012, an estimated 98.5 million US adults (42%) sought Web-based health information, 15.8 million (6.7%) ordered a Web-based prescription, 10.8 million (4.6%) made Web-based appointments with their health care provider, 13.5 million (5.7%) emailed their health care provider, and 6.8 million (2.9%) used Web-based health chat groups. Approximately half (116.7 million, 49.7%) of US adults reported having at least one chronic condition, and 57.3 million (24.4%), 32.7 million (13.9%), and 26.9 million (11.4%) reported having one, two, and three or more chronic conditions, respectively. The prevalence of each condition varies from the most frequently reported hypertension (50.5 million, 21.5%) to the least reported weak or failing kidneys (3.9 million, 1.7%).

### Chronic Conditions and HIT Use

A comparison of HIT use by respondents with and without chronic conditions is shown in Table 1. Prevalence of HIT use among adults with one chronic condition (raw count 4147/8551, weighted percentage 48.54%) was significantly higher than among those with MCCs (3816/9637, 39.55%) and those with none of chronic condition (7254/16,337, 44.40%). Adults with one chronic condition were significantly more likely than those in the other two groups to use HIT to look up health information, make an appointment, and use health chat group, whereas adults with MCCs reported highest prevalence of HIT use for ordering prescription and emailing health providers. The HIT use among adults varied by health conditions, ranging from 24.8% of respondents with stroke to 48.7% with asthma (data not provided in this paper).

**Table 1:** Weighted percentage of persons who had used health information technology by chronic condition groups.

Health information technology use variables	All, % (N^b^=34,525)	No chronic condition, % (N^b^=16,337)	One condition, % (N^b^=8551)	MCCs^a^, % (N^b^=9637)	Chi-square	*P* value
Any health information technology use	44.2	44.4	48.5	39.6	141.3	<.001
Looked up health information	42.0	42.4	45.9	37.2	133.5	<.001
Ordered prescription	6.7	4.7	8.4	9.0	218.8	<.001
Made appointment	4.6	4.4	5.4	4.3	15.5	.02
Emailed health provider	5.7	5.3	6.3	6.4	15.0	.02
Used health chat groups	2.9	3.0	3.2	2.5	8.5	.07

^a^MCCs: multiple chronic conditions.

^b^N: raw count.

**Table 2:** Comparison of characteristic between health information technology (HIT) users and nonusers among those who had at least one chronic condition in the past 12 months: weighted percentage and 99.5% CI.

Sociodemographic and health characteristics		All, % (N=34,525)	Did not use health information technology, % (99.5% CI) (N=20,178)	Used health information technology, % (99.5% CI) (N=14,347)
**Age (in years)**				
	18 to 29	8.6	7.4 (6.6-8.2)	10.2 (9.2-11.3)
	30 to 39	9.9	7.4 (6.8-8.2)	13.1 (12.2-14.0)
	40 to 49	16.0	13.6 (12.8-14.5)	19.1 (18.0-20.2)
	50 to 64	34.8	32.1 (30.9-33.4)	38.2 (36.7-39.7)
	65 to 74	16.9	19.2 (18.3-20.2)	14.0 (13.1-14.9)
	75+	13.7	20.2 (19.2-21.3)	5.4 (4.8-6.0)
**Gender**				
	Male	46.2	49.0 (47.8-50.2)	42.6 (41.1-44.1)
	Female	53.8	51.0 (59.8-52.2)	57.4 (55.9-58.9)
**Ethnicity**				
	Hispanic	10.5	13.1 (12.3-14.0)	7.1 (6.3-7.9)
	Non-Hispanic white	72.2	66.6 (65.3-67.9)	79.3 (78.1-80.5)
	Non-Hispanic black	12.6	15.4 (14.5-16.4)	9.1 (8.3-10.0)
	Non-Hispanic Asian	3.8	3.9 (3.4-4.4)	3.7 (3.2-4.3)
	Non-Hispanic all other race	0.9	1.0 (0.7-1.4)	0.8 (0.6-1.1)
**Education**				
	Less than high school	15.2	23.7 (22.6-24.8)	4.4 (3.8-5.0)
	High school graduate and some college	59.4	61.8 (60.6-63.0)	56.4 (54.8-57.9)
	Bachelor’s degree	15.6	9.3 (8.5-10.1)	23.6 (22.4-24.9)
	Master’s degree or higher	9.8	5.2 (4.6-5.8)	15.7 (14.6-16.8)
**Employment**				
	Not employed	44.9	54.5 (53.1-55.8)	31.5 (30.1-33.0)
	Employed	55.1	45.5 (44.2-46.9)	68.5 (67.0-69.9)
**Income (in US$)**				
	Up to 14,999	22.8	26.8 (24.8-28.9)	19.7 (18.3-21.1)
	15,000 to 34,999	24.2	32.3 (30.4-34.3)	24.2 (22.6-25.8)
	35,000 to 54,999	21.8	21.4 (19.7-23.2)	21.8 (20.2-23.4)
	55,000 to 74,999	14.0	9.3 (8.2-10.6)	14.0 (12.8-15.3)
	75,000 and higher	20.4	10.1 (8.7-11.7)	20.4 (18.7-22.1)
**Marital status**				
	Not in relationship	38.3	43.0 (41.7-44.4)	32.3 (31.0-33.7)
	In relationship	61.7	57.0 (55.6-58.3)	67.7 (66.3-69.0)
**Body mass index**				
	Up to 18.49	1.2	1.6 (1.3-2.0)	0.7 (0.5-1.0)
	18.5 to 24.9	25.6	25.0 (23.9-26.1)	26.3 (25.0-27.7)
	25-29.9	34.4	33.7 (32.5-34.9)	35.3 (34.0-36.6)
	30 and more	38.8	39.6 (38.5-40.8)	37.7 (36.3-39.0)
**Health status**				
	Very good to excellent	44.8	38.2 (37.1-39.3)	53.2 (51.7-54.7)
	good	33.4	34.6 (33.4-35.8)	31.8 (30.5-33.2)
	poor to fair	21.8	27.2 (26.1-28.4)	15.0 (14.0-16.1)

### Characteristics Associated With HIT Use Among Adults With Chronic Conditions

The characteristics related to HIT use among adults with at least one chronic condition are presented in Table 2. We found that HIT users significantly differed from nonusers with regard to sociodemographic and health characteristics. Compared with HIT nonusers, HIT users were significantly more likely to be under the age of 65 years, female, non-Hispanic white, with education level of bachelor’s degree or higher, having annual income of US $55,000 or higher, currently employed, and in a relationship. HIT users were significantly less likely than nonusers to report higher BMI level (≥30) and poorer self-rated health status (≤good).

### Potential Predictors of HIT Use

When adding the chronic condition status as an independent variable in the logistic regression model, the finding shows that higher prevalent HIT use is more likely to be reported by adults with one chronic condition (odds ratio, OR 1.55, 99.5% CI 1.44-1.68, *P*<.001) or with MCCs (OR 1.81, 99.5% CI 1.64-2.01, *P*<.001) than those with none of the 10 chronic condition. Table 3 presents results of the logistic regression analyses examining factors associated with HIT use by persons with none of the chronic conditions, one chronic condition, and MCCs. Overall, the significant predictors of HIT use were similar across all the three chronic condition groups. Specifically, after adjusting for all of the sociodemographic and health factors, those who were relatively younger, female, non-Hispanic white, with comparatively higher education level, and higher income level were significantly more likely to be HIT users. The OR differences varied in ±1 range for most of the predictors between those with chronic conditions and MCCs.

**Table 3:** Factors associated to health information technology (HIT) use among respondents with none, one chronic condition, and multiple chronic conditions (MCCs): weighted logistic regression model results.

Independent variable		With one chronic condition, adjusted OR^b^ (99.5% CI) N=8551	With MCCs^a^, adjusted OR (99.5% CI) N=9637	With no chronic conditions, adjusted OR (99.5% CI) N=16,337
**Age (in years)**				
	18 to 29	1.00	1.00	1.00
	30 to 39	0.95 (0.61-1.49)	0.61 (0.27-1.34)	0.77 (0.62-0.95)
	40 to 49	0.66 (0.42-1.04)	0.46 (0.22-0.98)	0.68 (0.55-0.85)
	50 to 64	0.51 (0.33-0.80)	0.40 (0.19-0.85)	0.54 (0.42-0.69)
	65 to 74	0.37 (0.21-0.65)	0.25 (0.12-0.55)	0.44 (0.26-0.76)
	75+	0.12 (0.03-0.45)	0.11 (0.04-0.32)	0.19 (0.04-0.91)
**Gender**				
	Male	1.00	1.00	1.00
	Female	1.92 (1.49-2.46)	2.21 (1.63-3.00)	2.25 (1.62-2.63)
**Ethnicity**				
	Hispanic	1.00	1.00	1.00
	Non-Hispanic white	1.93 (1.36-2.73)	1.95 (1.24-3.06)	1.58 (1.28-1.96)
	Non-Hispanic black	1.18 (0.76-1.83)	1.09 (0.62-1.91)	0.90 (0.68-1.20)
	Non-Hispanic Asian	0.93 (0.52-1.66)	1.04 (0.47-2.30)	1.20 (0.84-1.71)
	Non-Hispanic all other	1.39 (0.37-5.18)	2.00 (0.57-6.98)	1.01 (0.44-2.33)
**Education**				
	Less than high school	1.00	1.00	1.00
	High school graduate and some college	3.02 (1.71-5.35)	4.28 (2.38-7.68)	2.79 (2.07-3.787)
	Bachelor’s degree	6.88 (3.73-12.72)	12.66 (6.56-24.44)	5.89 (4.21-8.25)
	Master’s degree or higher	9.89 (5.00-19.57)	13.18 (6.55-26.51)	7.57 (5.11-11.20)
**Employment**				
	Not employed	1.00	1.00	1.00
	Employed	0.88 (0.47-1.64)	1.00 (0.56-1.78)	1.02 (0.65-1.61)
**Income (in US$)**				
	Up to 14,999	1.00	1.00	1.00
	15,000 to 34,999	1.05 (0.73-1.50)	0.88 (0.59-1.31)	1.05 (0.85-1.29)
	35,000 to 54,999	1.30 (0.90-1.87)	1.08 (0.71-1.64)	1.28 (1.01-1.61)
	55,000 to 74,999	1.46 (0.89-2.40)	2.02 (1.24-3.31)	1.35 (1.01-1.82)
	75,000 and higher	2.13 (1.34-3.37)	1.86 (1.09-3.18)	1.89 (1.39-2.56)
**Marital status**				
	Not in relationship	1.00	1.00	1.00
	In relationship	1.21 (0.94-1.55)	1.28 (0.95-1.73)	1.07 (0.91-1.25)
**Body mass index**				
	Up to 18.49	0.90 (0.29-2.79)	0.34 (0.07-1.74)	0.87 (0.45-1.70)
	18.5 to 24.9	1.00	1.00	1.00
	25-29.9	1.10 (0.80-1.52)	1.12 (0.73-1.73)	0.87 (0.73-1.03)
	30 and more	1.01 (0.76-1.35)	0.94 (0.62-1.41)	0.90 (0.74-1.11)
**Health status**				
	Very good to excellent	1.00	1.00	1.00
	Good	0.95 (0.72-1.25)	0.99 (0.72-1.36)	1.06 (0.86-1.31)
	Poor to fair	1.11 (0.73-1.70)	0.93 (0.61-1.4)	1.31 (0.87-1.98)

^a^MCCs: multiple chronic conditions.

^b^OR: odds ratio.

## Discussion

### Principal Findings

Our findings show that HIT use is relatively common among people with chronic conditions, ranging from about 40% of those with MCCs, to 49% of those with one chronic condition. The number of HIT users is expected be even higher nowadays with the increasing adoption of electronic health record (EHR) systems since the passage of the Health Information Technology for Economic and Clinical Health (HITECH) provisions of the American Recovery and Reinvestment Act (ARRA) of 2009 [18-20]. Of the five types of HIT use that were assessed, seeking Web-based health information was the most frequently reported use among all adults. This finding resonates with other reports that show health consumers are increasingly relying on the Internet for health information [9,10]. Among adults with one or more chronic condition, ordering Web-based prescriptions is the second most prevalent type of HIT use, with nearly 1 in 10 adults using the Internet to order prescriptions via a patient portal or pharmacy website. Recent research suggests that Web-based patient portal use may arguably be associated with better medication adherence, improved health care quality, and favorable patient outcomes [21,22]. Given that medication adherence is critical for chronic disease management, interventions including Internet-based approach that promote medication adherence are worth exploring [23]. Use of HIT to make appointments, email health care providers, and participate in chat groups for health topics was less prevalent. Although there has been little research to explain why the use of HIT for those other purposes is much lower, usability, availability, and accessibility of HIT functions, as well as health literacy could be some reasons [24,25]. Additionally, some adults with chronic conditions may face different difficulties accessing health care services, resulting in lower use of different kinds of HIT [26]. How the nature of a disease, severity and prevalence of the chronic conditions, and health care access affect HIT use warrants further examination.

We found that overall HIT use significantly differed among adults with or without chronic conditions, those with one chronic condition being the most active HIT users, those with MCCs the least, and those with none of the 10 chronic conditions falling in between. Our findings based on the multivariate regression models suggest that socioeconomic factors may have more influence on HIT use than health-related characteristics because the same sociodemographic factors were predictive of HIT use across all three of our study groups (adults with no chronic conditions, one chronic condition, and with MCCs). Specifically, consistent with the findings of previous studies on digital divide [8,27-29], we found that that across all three groups, HIT users were more likely than nonusers to be younger, female, non-Hispanic white, with comparatively higher education level, and with higher income level.

The lower use of HIT among adults with MCCs than those with one or no chronic condition may be explained by differences in the sociodemographic profile of each group. Whereas prevalence of MCCs varies by age, gender, and race or ethnicity, older age might be the key factor related to the lower use of HIT by adults with MCCs. First of all, for both genders, adults with MCCs are more likely to be older (aged ≥65 years) than those with only one or no chronic conditions [30,31]. Considering the rates of HIT use reportedly being significantly lower among the age groups 65 or older compared with the younger age groups [32], it is not surprising to find less prevalent HIT use among our MCCs respondents. The variation of gender and race or ethnicity might be outweighed by the impact of older age among those with MCCs [33]. This may further explain why HIT use is less among people with MCCs. In addition, adults with chronic conditions are reported more likely to have lower educational attainment and income [34,35]. Education and income factors are also related to health literacy [36], which in turn can have an impact on HIT use [24]. Aforementioned observations suggest that adults with MCCs are more likely than those with one or no conditions to be racial minorities, older, less educated, and with lower income; it is reasonable to expect lower HIT use in the MCCs group based on previous research. Regardless of which socioeconomic factors have more influence on HIT use, the above finding implies that educational materials or interventions to promote HIT use among those with chronic conditions must take into account socioeconomic factors that influence use. For instance, efforts should be made to help older adults and ethnic or racial minorities improve their abilities to navigate and utilize the Internet and recognize dependable Web-based sources so that they may increase their trust in its use, thereby increasing satisfaction with their own ability to seek and use sources of health information [37].

### Limitations

This study has a number of strengths, including using a dataset with a good response rate and a large sample drawn from a representative nationwide survey. Nonetheless, this study was subject to a few limitations. First, NHIS information was collected via self-report and the questions relating to health conditions and HIT use examined the participant’s experience in the previous 12 months; hence, the study findings are potentially subject to recall bias and social desirability bias. Second, because of the nature of the cross-sectional study design, it is not possible to draw conclusions about probable causal pathways between the two explored variables (eg, chronic conditions and computer use), and therefore, the study findings should be interpreted with caution. These limitations should be balanced against the strengths of the study, including the large sample size and representativeness of the US population.

### Conclusions

Our study provides a snapshot of HIT use among those with chronic conditions and potential factors related to such use. Our study suggests that HIT may serve as an alternative to more traditional methods of obtaining health information or communicating directly with health care providers, which in turn may help those with chronic conditions to better manage their illness over the long term. However, clinical care and public health communication efforts attempting to leverage more HIT use should acknowledge differential HIT usage as identified in this study to better address communication inequalities and persistent disparities in socioeconomic status.

## Abbreviations

BMI: body mass index
CDC: Centers for Disease Control and Prevention
COPD: chronic obstructive pulmonary disease
DHHS: Department of Health and Human Services
EHR: electronic health record
HIT: health information technology
HITECH: health information technology for economic and clinical health
MCCs: multiple chronic conditions
NHIS: National Health Interview Survey
NCHS: National Center for Health Statistics
OR: odds ratio
SPSS: Statistical Package for Social Sciences
